# Inflammaging in periodontal, periapical, and malignancy-associated disease: drivers of alveolar bone loss and repair

**DOI:** 10.1007/s00774-026-01706-2

**Published:** 2026-02-18

**Authors:** Meircurius Dwi Condro Surboyo, Kridtapat Sirisereephap, Tomoki Maekawa

**Affiliations:** 1 Center for Advanced Oral Science, Graduate School of Medical and Dental Sciences, Niigata University, Niigata, 951-8514 Japan; 2 Department of Oral Medicine, Faculty of Dental Medicine, Universitas Airlangga, Surabaya, 60132 Indonesia; 3 Faculty of Dentistry, Chulalongkorn University, Bangkok, 10330 Thailand

**Keywords:** Aging, Inflammaging, Periodontal, Periapical bone, Malignancy

## Abstract

**Background:**

Aging significantly impacts bone metabolism through altered osteoblast/osteoclast dynamics, reduced stem cell regeneration, and chronic inflammaging. This narrative review explores how these age-related changes influence alveolar bone loss and regeneration in the oral cavity.

**Methods:**

The review investigates key mechanisms—including immunosenescence and inflammasome activation—across three specific pathological contexts: (1) periodontitis, (2) periapical bone resorption, and (3) malignancy-associated osteolysis. Preclinical and clinical evidence were integrated to analyze the bone-immune equilibrium.

**Results:**

Aging was found to skew the immune environment, exacerbating bone destruction. The review identifies emerging immunomodulatory strategies to rejuvenate bone healing, such as targeting senescent cells (senolytics) and inflammatory cytokines to modulate the immune microenvironment.

**Conclusion:**

Addressing the unique challenges of the aging population is critical for regenerative dentistry. Future research must bridge current gaps to translate immunomodulatory insights into clinical therapies for improving alveolar bone regeneration in older patients.

## Introduction

Population aging has brought increased attention to the impact of age-related biological changes on oral health [1]. The alveolar bone is uniquely affected by both systemic aging processes and local pathological challenges. Conditions, such as periodontitis and endodontic infections (pulpal necrosis leading to periapical lesions), directly involve the inflammatory destruction of the alveolar bone, and these conditions often present with greater severity or slower healing in older adults [2]. Similarly, oral malignancies (such as oral squamous cell carcinoma or metastatic lesions in the jaw) can cause aggressive local bone resorption [3]. Therefore, understanding bone metabolism in the context of aging is critical for the development of regenerative therapies in dentistry and oral surgery.

Bone remodeling is a dynamic equilibrium between bone formation by osteoblasts and bone resorption by osteoclasts. This balance is regulated by various systemic hormones, local growth factors, and cytokine signals from the bone marrow and the immune system [4]. With aging, skeletal homeostasis becomes dysregulated; osteoblast activity and number tend to decline, whereas osteoclast activity can be relatively unopposed, tipping toward net bone loss [5]. Furthermore, aging bone marrow mesenchymal stem cells (also termed skeletal stem cells, SSCs) exhibit reduced osteogenic potential and a tendency to produce more pro-inflammatory factors [6]. At the same time, the aging immune system undergoes immunosenescence (functional decline of adaptive immunity) and develops a pro-inflammatory baseline state known as inflammaging, marked by elevated circulating cytokines, such as interleukin (IL)-6 and tumor necrosis factor (TNF)-α [7]. These systemic changes create an environment that exacerbates bone resorption and impairs bone regeneration.

In the oral cavity, alveolar bone is highly active and remodels in response to mechanical forces and inflammation. Alveolar bone loss is not an inevitable consequence of aging alone; however, age can amplify the responses to pathological insults. Older individuals have a higher prevalence of periodontitis and often present with cumulative attachment loss; however, age per se is considered a modifying factor rather than a primary cause of periodontal disease [8]. The interplay of aging with chronic oral infections and diseases is complex. For example, an older patient might exhibit a heightened inflammatory reaction to periodontal bacteria due to an “aged” innate immune system, yet simultaneously exhibit deficits in the resolution and healing phase [9]. Similarly, bone healing after dental implantation or endodontic surgery may be delayed in older patients owing to reduced osteoprogenitor cell activity and vascularity [10]. Oral malignancies typically occur in older patients who may have age-compromised bone quality, compounding the challenges of surgical reconstruction and regeneration after tumor resection [11].

In this review, we outlined the fundamental mechanisms by which aging affects bone metabolism at the cellular and molecular levels. We then delved into three specific clinical contexts—periodontitis, periapical lesions, and tumor-associated bone resorption—to discuss how age-related changes modify disease progression and the capacity for alveolar bone regeneration. We highlight osteoimmunological concepts, including immune cell aging and the chronic inflammation interface with osteoblast/osteoclast regulation in these diseases. In each scenario, preclinical (animal) models and clinical observations are used to illustrate the key points. Finally, we explore the emerging immunomodulatory and regenerative strategies for counteracting the detrimental effects of aging on bone healing. By emphasizing translational approaches, we aim to identify pathways that could be targeted to improve clinical outcomes in older patients with alveolar bone loss due to periodontal disease, endodontic pathology, or oral cancer. By understanding the nexus between bone biology and immunology in aging, we can better design therapies for regenerative dentistry that are effective across the lifespan.

## Methods

This narrative review is intended to provide a conceptual and mechanistic synthesis of how aging biology influences alveolar bone loss and regeneration. Literature was identified through iterative, topic-driven searches in commonly used biomedical databases, including PubMed/MEDLINE, Scopus, and Web of Science. Searches were conducted across multiple stages of manuscript preparation and were refined as key mechanistic themes emerged.

Search terms included combinations of “aging”, “periodontitis”, “apical periodontitis”, “periapical lesion”, and “oral cancer”. Reference lists of key articles were also reviewed to identify additional relevant studies.

Studies were selected based on conceptual relevance, mechanistic insight, and translational value, rather than on formal eligibility scoring. Human clinical and translational studies were prioritized when available, whereas animal and in vitro studies were used to support biological mechanisms.

## Mechanisms of aging in bone metabolism

Aging bones are characterized by a gradual shift toward decreased anabolic activity and increased catabolic activity [12]. At the cellular level, a reduction in the number and function of osteoblasts derived from mesenchymal stem cells (MSCs) occurs, as well as an accumulation of old osteocytes (terminally differentiated osteoblasts embedded in the bone) that may become dysfunctional [13]. Concurrently, osteoclast precursors in aged individuals often experience an altered signaling milieu that favors bone resorption. Coupling between bone formation and resorption is less tightly regulated by age. Key mechanisms underlying these changes include cellular senescence, stem cell exhaustion, hormonal alterations, and chronic low-grade inflammation [14, 15].

### Decline in osteogenic capacity

MSCs and skeletal progenitor cells from aged bone marrow or periodontal tissues show diminished proliferative and osteogenic capacities compared with those from young individuals. For instance, human periodontal ligament stem cells isolated from older adults have lower growth and differentiation potential than those isolated from younger donors [16]. Similarly, aged skeletal stem cells in mice generate fewer osteoblasts and skew toward adipogenic or fibrotic lineages that secrete pro-inflammatory cytokines [6]. The intrinsic aging of osteoprogenitors leads to reduced bone formation. Aged osteoblasts also show altered gene expression, including increased levels of Wnt signaling inhibitors and decreased production of bone matrix proteins [17]. Changes in hormones exacerbate this imbalance. Estrogen normally promotes osteoprotegerin (OPG) and restrains receptor activator of nuclear factor kappa-Β ligand (RANKL), thus suppressing osteoclastogenesis; with menopause, loss of estrogen tilts the RANKL/OPG ratio upward, accelerating bone resorption [18]. Postmenopausal changes have been linked to the loss of trabecular bone density in the jawbones. Therefore, the aged skeletal system is less capable of forming new bone and is more prone to bone loss under homeostatic conditions.

### Osteoclast activity and bone resorption

The population of osteoclast precursors in the bone marrow does not necessarily increase with age; however, they respond to a more inflammatory environment and may become overactive relative to the reduced osteoblast population [19]. The net result is often an increase in bone resorption or an impaired ability to refill the resorptive cavities. Chronic oxidative stress in the aged bone niche can stimulate osteoclastogenesis by activating redox-sensitive pathways (e.g., nuclear factor-κB) [19]. Inflammaging contributes significantly here: higher circulating levels of TNF-α, IL-1β, IL-6, and other cytokines in older adults can directly or indirectly (through stromal cells) enhance RANKL-mediated differentiation of osteoclasts [20]. Furthermore, old bone marrow stromal cells produce fewer osteoblast-supporting factors and more pro-osteoclast factors [6]. Ambrosi et al*.* [6] have demonstrated that aged skeletal stem cells in mice create an “inflammatory degenerative niche” by secreting cytokines that promote osteoclast activity and myeloid cell skewing. This helps explain why aged bones are prone to osteopenia and heal more slowly, and why the regenerative cells are fewer in number and actively induce a bone-degrading environment.

### Cellular senescence and senescence-associated secretory phenotype

The accumulation of senescent cells is a hallmark of tissue aging. Osteocytes are long-lived cells that develop senescence markers in old age [21]. Senescence-Associated Secretory Phenotype (SASP) that releases pro-inflammatory and tissue-degrading molecules. In aged murine alveolar bone, osteocytes have been shown to upregulate SASP factors, including IL-6, IL-17, and matrix metalloproteinase-13 [21]. SASP factors have been shown to promote pro-resorptive signaling and impair osteogenic activity in aged murine models, suggesting a potential contribution to bone loss. Aquino-Martinez et al*.* have found that osteocytes from old mice (≈20–22 months) had higher DNA damage and p16^Ink4a^ expression, and their secretory profile potentiated inflammation. For example, conditioned media from senescent osteocytes caused increased IL-1β and IL-6 production when young bone cells were exposed to bacterial toxin (lipopolysaccharide) [21]. Notably, this SASP milieu impedes the migration and differentiation of progenitor cells, indicating the direct inhibition of regeneration [22]. These effects can be mitigated by targeting the p38 MAPK pathway, a stress-induced kinase upregulated in aged osteocytes, and using a p38 inhibitor to reverse some of the inflammatory gene expression and functional deficits in vitro [21]. This suggests that cellular senescence is not merely a bystander effect of aging but also an active driver of bone metabolic imbalance, which is supported by the observation that clearing senescent cells or suppressing their SASP can rejuvenate bone formation in experimental models.

### Immunosenescence and inflammaging

Aging of the immune system involves two seemingly paradoxical aspects: a decline in adaptive immunity (immunosenescence) and an increase in basal inflammatory activity (inflammaging). Immunosenescence includes reduced lymphocyte development and function, particularly T cell and B cell responsiveness, as well as functional alterations in innate immune cells [23]. In the context of bones, immunosenescence may impair the body’s ability to clear infections or resolve inflammation, leading to persistent low-grade inflammation that harms the tissues. In the bone, this is reflected by elevated levels of bone-resorbing cytokines (such as IL-1 and TNF) and chemokines that recruit osteoclast precursors [24]. A recent review by Zhou et al*.* [7] reported that in periodontitis (an aging-associated condition), “inflammaging” is marked by abnormal polarization of macrophages to a pro-inflammatory (M1) state and increased systemic cytokines, whereas immunosenescence manifests as poorer pathogen clearance and an accumulation of memory T and B cells that can produce inflammatory signals. Collectively, these processes significantly affect alveolar bone turnover with age. For example, toll-like receptor signaling may become overactive in the aged periodontium, leading to increased inflammatory osteoclast activation upon microbial challenge [25]. These mediators sustain osteoclastogenesis and inhibit osteoblasts, contributing to bone loss.

### Inflammasome activation

The inflammasome, particularly the NLR family pyrin domain containing 3 (NLRP3) inflammasome, is a cytosolic complex in immune cells that controls the activation of caspase-1 and the maturation of IL-1β and IL-18, which are key inflammatory cytokines for bone resorption. Aging is associated with a primed inflammasome state, and oxidative stress and endogenous danger signals trigger NLRP3 expression more readily in aged cells. This has implications for bone: IL-1β is a potent driver of osteoclast differentiation and bone loss. Activation of the NLRP3 inflammasome has been shown to promote IL-1β–dependent osteoclastogenesis and alveolar bone resorption in animal models, whereas human studies report elevated inflammasome-related markers in diseased tissues [26]. Excess reactive oxygen species in aging tissues can activate the reactive oxygen species-NLRP3-IL-1β axis, leading osteoblasts to undergo a form of pyroptosis that undermines bone formation [27]. Moreover, a recent study in aged mice reported that the transcription factor forkhead box O1 (involved in cellular stress responses) drives NLRP3 inflammasome-mediated bone loss and that forkhead box O1 deficiency protects older mice from alveolar bone resorption, suggesting that targeting this pathway can ameliorate age-related inflammatory bone damage [28]. Thus, the inflammasome represents a molecular bridge between aging and bone catabolism through IL-1–driven osteoclast activation.

### Interactions in the bone marrow niche

The bone microenvironment comprises bone cells, marrow cells (hematopoietic cells and others), and vasculature. Aging causes a shift in this niche; hematopoietic stem cells in older adults are skewed toward myeloid lineages (producing more osteoclast precursors and inflammatory cells and fewer lymphocytes). Aged mesenchymal cells secrete factors that drive myeloid skewing [6]. Evidence that normal bone-protective interactions are impaired with age is also available. For example, megakaryocytes (platelet-producing cells) in the marrow usually help maintain bone mass by stimulating osteoblast proliferation and restraining osteoclasts; aging disrupts this crosstalk, contributing to bone loss [29]. Reduced angiogenesis is another factor; the microvascular supply in the bone deteriorates with age, which can limit the delivery of oxygen/nutrients and the necessary cells for regeneration. All these changes coalesce into an “aged bone niche” that is less hospitable to regeneration and more permissive of chronic inflammation and bone degradation.

### Conceptual framework for aging-related alveolar bone loss

**Fig. 1:**
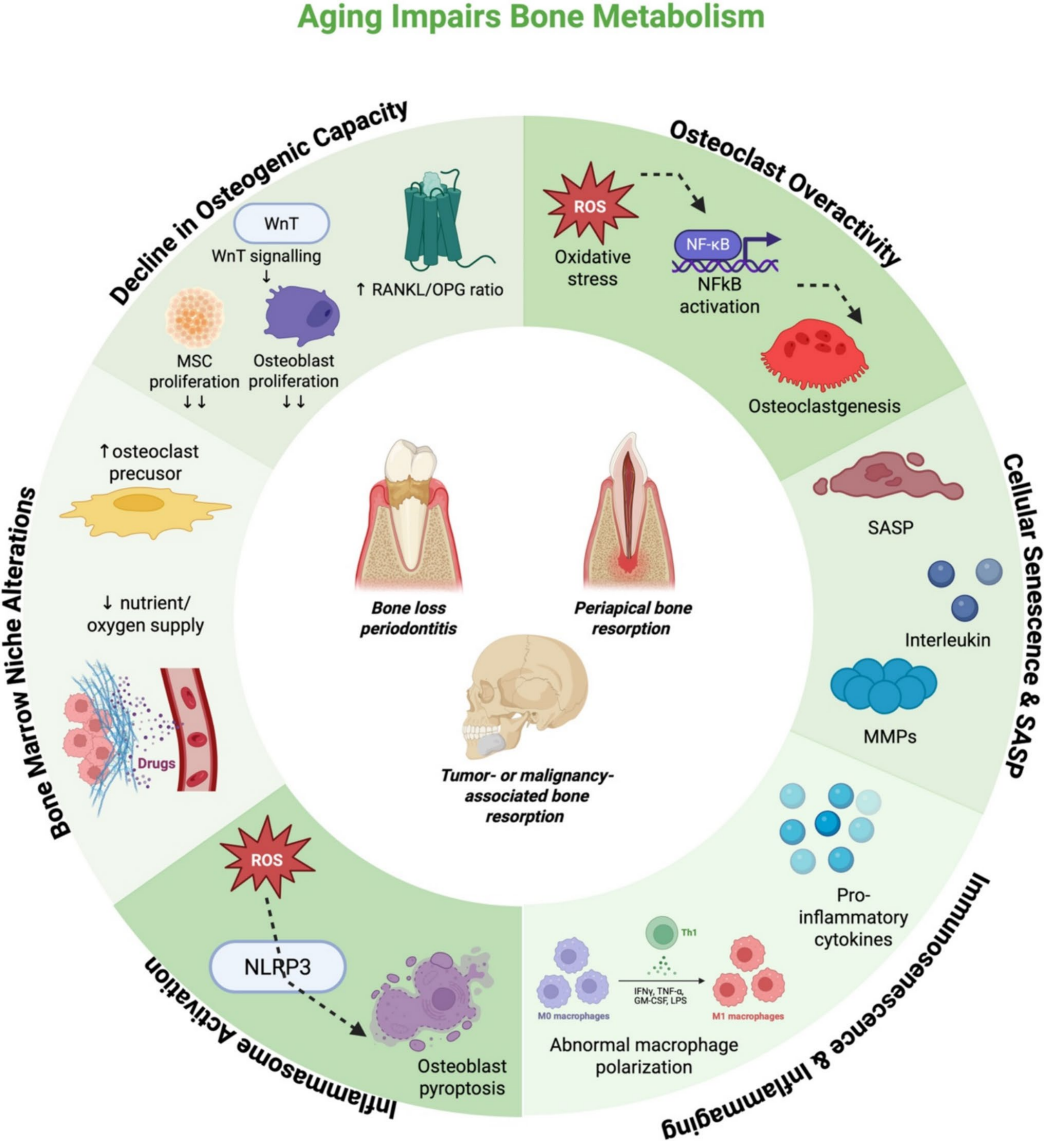
Mechanisms of aging in bone metabolism. Aging disrupts skeletal homeostasis through a combination of reduced osteogenic capacity, increased osteoclast reactivity, accumulation of senescent osteocytes with SASP secretion, immunosenescence with chronic inflammaging, NLRP3 inflammasome activation, and detrimental alterations in the bone marrow niche. Taken together, these mechanisms shift the balance toward higher bone resorption and impaired regeneration. SASP, Senescence-Associated Secretory Phenotype; NLRP3, NLR family pyrin domain containing 3

As summarized above, aging alters bone metabolism through intrinsic cellular changes (senescence and reduced stem cell function), hormonal and molecular shifts (e.g., decreased estrogen and increased reactive oxygen species), and immune system remodeling (immunosenescence with concomitant inflammaging and inflammasome activation) (Fig. 1). These changes do not uniformly cause disease; rather, they modify host responses, rendering alveolar bone more vulnerable to inflammatory insults and less capable of efficient regeneration following injury. The following sections examine how these aging-related mechanisms operate in specific oral disease contexts.

Apparent inconsistencies in the reported effects of aging on alveolar bone loss largely reflect differences in disease stage, experimental context, outcome measures, and host characteristics. In acute or short-term experimental models, particularly those assessing early inflammatory responses, aged hosts often exhibit reduced immune cell recruitment, attenuated peak inflammatory signaling, and lower short-term osteoclast activation, consistent with features of immunosenescence.

In chronic disease settings and clinical observations, aging is associated with persistent low-grade inflammation, impaired resolution of inflammatory responses, accumulation of senescent cells, and reduced osteogenic and angiogenic capacity. These features may not increase peak bone resorption but instead promote prolonged inflammation, defective repair, and greater cumulative bone loss over time. Consequently, studies focused on early lesion size or peak bone resorption may report an attenuated destructive response in aged hosts, whereas studies in which researchers evaluate disease persistence, cumulative bone loss, or regenerative outcomes consistently report impaired healing and worse long-term outcomes with aging.

Recognizing these distinctions offers a coherent framework in which aging is seen not as uniformly protective or destructive, but as a modifier that alters the temporal dynamics of inflammation and the capacity for tissue resolution and regeneration.

Although the aging-related mechanisms discussed above have been characterized in long bones and bone marrow, alveolar bone represents a distinct biological compartment. Unlike the appendicular and axial skeleton, alveolar bone is developmentally linked to tooth eruption, exhibits a high turnover rate throughout life, is functionally integrated with the periodontal ligament, and is continuously exposed to microbial and mechanical challenges. Consequently, mechanisms derived from general skeletal aging studies cannot be directly extrapolated to the jaw without contextual consideration. In this review, findings from long-bone or marrow-based studies are therefore discussed as mechanistic frameworks, whereas conclusions regarding disease progression and regeneration are grounded in jaw- and alveolar-specific evidence whenever available.

## Disease-specific discussions

Major clinical modifiers that commonly coexist in older adults, including sex differences, metabolic disorders (particularly diabetes mellitus), smoking status, polypharmacy, and exposure to antiresorptive or anticancer therapies must be considered when interpreting aging-related effects in the following disease contexts. These factors interact with aging-related immune and skeletal changes and substantially influence the magnitude, trajectory, and reversibility of alveolar bone loss.

### Periodontitis and alveolar bone loss in the aged

The hallmark of periodontitis is the loss of attachment, including breakdown of the periodontal ligament and resorption of the alveolar bone, ultimately leading to tooth loosening. In older adults, periodontitis tends to present as a “high-inflammation, low-repair” state in the periodontium.

One key aspect is the contribution of the aged immune response to periodontal bone loss. Periodontal lesions are characterized by the infiltration of neutrophils, macrophages, T cells, and B cells that produce an array of cytokines and proteases. In younger individuals, although these mediators cause bone resorption, the capacity for resolution and tissue repair if the infection is controlled exists. However, the resolution may be impaired in older individuals. Studies have shown that *immunosenescence* skews the response; for example, neutrophils from aged mice have reduced chemotaxis toward bacterial chemoattractants but paradoxically exhibit a hyperactive phenotype in situ, releasing more superoxide radicals and neutrophil extracellular traps that can damage tissue [30]. Magne et al. [30] have reported that in a ligature-induced periodontitis model, aged mice (versus young mice) had greater alveolar bone loss, and their neutrophils displayed excessive reactive oxygen species generation and NETosis despite sluggish migration. These “hyperfunctional” neutrophils likely contribute to collateral tissue damage in periodontal lesions of older hosts.

Macrophages and lymphocytes behave differently in the aging periodontium. Aged macrophages often exhibit an M1 pro-inflammatory bias and decreased clearance of apoptotic cells, thereby prolonging inflammation [7]. Aging adaptive immunity features an expansion of memory T cells and less naive T cell output. Th17 cells, which secrete IL-17A and RANKL, both potent stimulators of osteoclasts, are increased in periodontitis. Studies have shown that reducing Th17 activity can limit alveolar bone resorption [31]. In older individuals, an elevated basal IL-17 environment has been observed in the bone tissue, suggesting that inflammaging may tilt T cell responses toward osteoclast-activating profiles [21]. Moreover, B cells in periodontal lesions can differentiate into plasma cells that produce RANKL. With age, a higher proportion of differentiated B cells may infiltrate the gingiva [7].

The exaggerated production of osteoclastogenic signals is a direct consequence of these immune changes. Gingival fibroblasts and periodontal ligament cells from older patients may produce more pro-inflammatory mediators in response to bacterial lipopolysaccharide than those from younger cells. For instance, aged gingival fibroblasts show a five-fold decrease in collagen synthesis and increased production of inflammatory prostaglandin E2 and IL-1β when challenged with bacterial components [5]. These cytokines (prostaglandin E2, IL-1β) and others, such as IL-6 and TNF-α, amplify RANKL expression on osteoblasts/stromal cells or are released in soluble form by T and B cells, driving local osteoclast differentiation.

Compelling evidence for the intersection between aging and periodontal inflammation comes from molecular analysis of human periodontal tissues stratified by age. A study by Teixeira et al*.* measuring inflammatory cytokine expression in the gingiva and reported that older patients (> 60 years) with chronic periodontitis had significantly higher levels of IL-1β, IL-6, and TNF-α in their lesions than in middle-aged adults [32]. This aligns with the concept that “inflammaging” upregulates these bone-resorptive cytokines. Another noteworthy finding is the role of senescent cells in periodontal disease progression: alveolar bone osteocytes showing senescence markers were more prevalent in old mice with periodontitis, and their SASP factors (IL-6, IL-17, etc.) likely fueled further bone destruction. Aquino-Martinez et al*.* have demonstrated that the accumulation of senescent osteocytes in the periodontium of old mice exacerbates chronic inflammation and reduces bone regeneration, thereby accelerating periodontal breakdown [21]. This raises the intriguing possibility that senolytic strategies have been shown to improve bone parameters in aged animal models; whether similar benefits can be achieved in alveolar bone regeneration in humans remains to be established.

From a clinical perspective, older persons with periodontitis may experience more rapid radiographic bone loss and a blunted healing response to conventional therapy. For example, periodontal regenerative procedures (such as guided tissue regeneration or bone grafts) can be successful in older patients; however, healing may be slower and less predictable. The basic biological reasons include reduced cell turnover in the periodontal ligament, diminished angiogenesis in the bone, and perhaps a dominance of pro-inflammatory immune cells that do not easily switch to a healing phenotype. Additionally, the periodontal ligament in older adults has fewer cells and a more irregular structure. Mechanical loading on the periodontium (e.g., chewing forces) in older individuals might cause micro-damage that is repaired more slowly, thereby compounding disease susceptibility.

Some animal studies have suggested that aged subjects may sometimes exhibit less acute bone resorption than young subjects in certain models of periodontitis because of weaker immune activation. For instance, a mouse study of periodontal pathogen infection reported that young mice showed a strong immune response with significant bone loss, whereas older mice had dampened immune cell infiltration and showed lower peak bone resorption; however, they also failed to clear the infection effectively, leading to a chronic low-grade lesion [33]. This highlights that aging can both attenuate and aggravate aspects of the disease. Initial acute responses may be blunted (reducing early tissue breakdown), yet the inability to resolve inflammation can result in prolonged disease activity and cumulative bone damage over time.

In summary, periodontitis in older individuals is influenced by immunosenescence (reduction in protective immune functions), inflammation (increase in destructive inflammatory signaling), and the presence of senescent cells in periodontal tissues. All these factors converge to shift the balance toward bone resorption and away from regeneration. Effective management of periodontitis in older patients may require mechanical removal of bacterial plaque and adjunctive strategies to modulate the host response, as discussed in later sections on immunomodulatory therapies.

### Periapical bone resorption due to pulp infection (endodontic lesions)

In chronic periapical periodontitis, bone metabolism and immune responses are closely intertwined. These lesions result from the host’s immune reaction to a bacterial infection, which spreads from the root canal into the periapical tissues, causing localized bone resorption [34]. The effects of aging on periapical lesion development and healing are highly pertinent to endodontic outcomes in older patients.

In the acute phase of pulp infection, neutrophils and macrophages flood the periapical area, releasing enzymes and reactive oxygen species that begin to erode the bone [35]. Over time, chronic granulomatous tissue forms, containing macrophages, lymphocytes, and often an epithelial component. Bone resorption at the apex is driven by many of the same mediators as in periodontitis, such as IL-1β, TNF-α, IL-6, and prostaglandins, which stimulate osteoclast formation [36]. Aging can influence this process in several ways, including differences in immune cell function, inflammatory milieu, and regenerative capacity of the bone.

Human clinical data suggest that older individuals have a more intense expression of inflammatory mediators in chronic apical lesions. In 2021, Teixeira et al*.* compared the immunohistochemistry of cytokines in chronic apical periodontitis lesions in older patients (> 60 years) and younger adults [32]. The results showed significantly higher levels of IL-1β, IL-6, and TNF-α in lesions of the older group. These cytokines are central to periapical bone resorption; IL-1β in particular has long been known to directly induce osteoclast activation in apical periodontitis. The heightened presence of these cytokines in the lesions of older patients aligns with the concept of inflammaging: older individuals mount a more pro-inflammatory chronic response, potentially leading to larger or more persistent lesions. This also raises concerns that the same factors that impede periodontal healing may hinder the resolution of apical lesions.

Animal studies have also provided further insights. An intriguing mouse model compared apical periodontitis development in young and older mice (e.g., young adult vs. middle-aged mice) by inducing pulp exposure and infection. Older mice developed smaller periapical lesions than younger mice, accompanied by fewer neutrophils and osteoclasts [33]. Young mice show robust neutrophil infiltration and osteoclastic activity, causing more extensive bone destruction [37]. This suggests that the attenuated immune response in older mice leads to less acute bone resorption. Translating this to humans, older patients might not experience pronounced pain or swelling (signs of acute apical abscess) owing to a blunted response; however, they could develop a chronic apical granuloma that remains unresolved.

The capacity of periapical bone regeneration after treatment is also affected by age. Successful endodontic treatment removes the source of the infection, allowing the inflammatory drive for bone resorption to cease and bone healing to commence. Clinical follow-up studies using radiography or cone beam computed tomography have identified age as a factor that affects healing speed. A recent cone beam computed tomography-based retrospective analysis of large apical lesions (10–15 mm) treated non-surgically revealed that although approximately 76% of the lesions healed completely, the time to radiographic healing was significantly longer in older patients. Specifically, an increase in patient age was associated with a prolonged healing time, with many older patients taking well beyond 18 months to fully ossify, whereas lesions in younger patients often healed between 12 and 18 months [38]. This study reported that “periapical lesions in older patients and larger areas of bone loss take longer to heal.”

In clinical practice, delayed periapical healing in older patients is further influenced by systemic metabolic conditions (such as diabetes mellitus), smoking status, and medication exposure, which independently affect angiogenesis, immune resolution, and osteogenic capacity beyond the effects of chronological aging alone.

Immunologically, older age may alter the profile of the cells that orchestrate periapical healing. Younger patients tend to mount a strong initial inflammatory response that transitions into a healing phase dominated by macrophages that shift to the M2 phenotype and recruit osteoblast precursors to lay down new bone. In older patients, there may be an imbalance in the presence of pro-inflammatory M1 macrophages or senescent immune cells. A recent concept in endodontic research is that inflammasome activity in periapical lesions contributes to bone destruction, and aging may upregulate inflammasome components, similar to periodontitis. For example, NLRP3 and caspase-1 have been shown to be active in human periapical tissues, and their blockade could reduce IL-1β levels and lesion size [39]. If older patients have a higher baseline inflammasome activation, they might experience more IL-1β-driven tissue breakdown in the chronic phase of apical periodontitis.

Another factor is the regenerative properties of the periapical periosteum and endosteum. These tissues contain stem cells that form the bone. Available studies report that periapical healing relies on mesenchymal stem cell recruitment from the bone marrow and perhaps the periodontal ligament [40]. Aging can reduce the number or osteogenicity of recruited cells. Additionally, repeated episodes of periapical inflammation can induce scarring or irreversible changes in the local microenvironment, making it less conducive to bone regeneration. Notably, clinical progression of periodontitis in older adults is strongly modified by sex-related hormonal status, smoking, glycemic control, and medication use, which can amplify or mask aging-associated immune and bone remodeling changes.

In summary, although the initial acute response to pulpal infection may be less intense in older individuals, the chronic phase of periapical lesions in older individuals tends to show higher pro-inflammatory cytokine levels and slower or incomplete bone repair. Clinically, endodontists should be aware that teeth in older patients may show persistent apical radiolucencies for longer post-treatment periods, necessitating extended observation periods. Strategies to improve healing include systemic or local adjuvants that enhance bone formation or modulate inflammation in older individuals. The interplay between aging and apical periodontitis underscores the importance of considering host factors in the prognosis and treatment planning for endodontic diseases.

### Tumor- or malignancy-associated bone resorption in the oral cavity

Malignant tumors involving the oral and maxillofacial regions frequently lead to destruction of the adjacent bone. Bone loss can be rapid and aggressive and is commonly associated with tumor-induced osteoclast activation and/or direct invasive growth. Although tumor-related bone resorption is principally dictated by the biology of the cancer, the context of aging is relevant because most patients with oral cancer or bone metastases are older adults. Age-related bone conditions (such as osteopenia or Paget’s disease) and immune changes may influence the interaction between the tumor and the bone microenvironment. Additionally, bone regeneration after tumor resection must contend with reduced healing capacity in older patients.

#### Local invasion by oral cancers

Because direct mechanistic studies of jaw-specific tumor invasion and metastasis are limited, the following discussion integrates available jaw-specific observations with hypotheses derived from systemic and long-bone metastasis models, which are presented as mechanistic frameworks rather than established jaw-specific pathways.

Oral squamous cell carcinoma (OSCC) is a common oral malignancy that often presents in the 6th to 8th decades of life. When OSCC is located in the gingiva or retromolar area, it can invade the underlying mandible or maxilla. Tumor invasion into bone has been described as occurring through a “vicious cycle” similar to mechanisms established in skeletal metastasis models; cancer cells stimulate osteoclasts to resorb bone, and the released growth factors from bone matrix in turn promote tumor growth. A recent clinical study analyzing OSCC with mandibular invasion reported that areas of bone invasion had significantly higher numbers of osteoclasts than non-invaded areas, and that OSCC cells at the bone front showed increased expression of RANKL and RANK. These findings suggest that OSCC cells may upregulate components of the RANK–RANKL pathway at the bone invasion front, which is consistent with the enhanced osteoclast activity observed in invaded regions [41].

From a molecular standpoint, OSCC and other cancers have been reported to express various osteolytic mediators, including parathyroid hormone-related peptide, IL-6, IL-8, vascular endothelial growth factor, and proteases, all of which have been implicated in promoting bone degradation in experimental and clinical studies. In skeletal metastases, the RANKL:OPG ratio in the bone often shifts drastically in favor of RANKL [42]. Although data specific to jaw metastases are limited, findings from long-bone metastasis models suggest that similar osteoclast-activating signaling pathways may be engaged following tumor colonization of jawbone. For example, breast cancer cells frequently secrete parathyroid hormone-related peptide in the bone environment, which binds to osteoblasts and stromal cells and triggers the upregulation of RANKL while downregulating OPG [43]. This mechanism has been well-documented as a driver of osteolytic metastases in long bones [44], and analogous processes may occur in the jaw, although direct experimental validation remains limited.

Distinguishing local OSCC-driven bone invasion from metastatic osteolysis in the jaw is crucial. Local invasion reflects direct tumor–bone interactions at the primary tumor margin and is influenced by local inflammatory, mechanical, and stromal factors specific to the oral microenvironment. In contrast, metastatic osteolysis involves hematogenous tumor dissemination, bone marrow colonization, and systemic tumor–bone signaling mechanisms, which are biologically distinct processes and far less common in the jaw.

#### Impact of aging on tumor–bone interactions

Older patients may experience more severe consequences of tumor-related bone loss for a few reasons. First, they may have a pre-existing low bone density or osteoporosis, which means that less reserve is available before a pathological fracture or tooth loss occurs from tumor osteolysis. Second, the ability of the peritumoral bone to mount reactive bone formation can be diminished. In younger patients, the host response to a tumor invading the bone sometimes includes laying down new bone at the margins; in older patients, this osteogenic response might be weaker, allowing more extensive infiltration. Immunosenescence may play a role in this process. Research is emerging on how an aging immune microenvironment facilitates cancer progression. In the bone microenvironment, age-related immune changes may be associated with reduced containment of tumor cells and prolonged osteoclast activity, although direct clinical evidence in the jaw remains limited, unchecked by immune regulation.

Many therapies for cancers that cause bone loss involve antiresorptive agents, which have implications for the aging population. These drugs are often administered to prevent skeletal events in metastatic cancer or multiple myeloma (median age ~ 70 years), curb osteoclast activity, and protect bone mass. For instance, clinical studies have shown that denosumab (a monoclonal antibody against RANKL) reduces tumor-associated osteolysis by inhibiting RANKL-mediated osteoclast activation though blocking the RANKL–osteoclast pathway [45]. Although beneficial for controlling bone destruction, their use in the jaw is tempered by the risk of medication-related osteonecrosis of the jaw, especially in older patients with comorbidities. Medication-related osteonecrosis of the jaw is a failure of bone healing that can occur spontaneously or after dental extraction when potent antiresorptives are used. This underscores that manipulating bone remodeling in the context of malignancy and aging is a double-edged sword: halting osteoclasts protects bone from cancerous destruction but may also impair normal regenerative turnover, particularly in alveolar bone regularly subjected to micro-injury and dental interventions.

#### Bone regeneration after tumor resection

In oral cancer therapy, a common scenario is surgical resection of a part of the jaw to remove the tumor, followed by efforts to restore the form and function of the jaw. Older patients also have disadvantages regarding regeneration. They often have slower wound healing, and autogenous bone graft quality may be compromised by age-related factors [46]. Additionally, radiation therapy, which is frequently used as an adjunct treatment in head and neck cancers, causes further damage to bone vascularity and cells, compounding the challenge of regeneration. The intersection of aging and radiation leads to a highly compromised healing environment, which can result in osteoradionecrosis if not carefully managed.

From a pathophysiological perspective, after tumor removal, the healing of bone defects depends on periosteal and marrow cells to produce new bone, as well as on angiogenesis to vascularize the regenerating tissue. In older patients, periosteal ossification tends to be less robust, and the periosteum itself may be less cellular. The growth factor levels that drive repair may be lower or less responsive in aged tissues.

In conclusion, malignancy-associated alveolar bone resorption is largely governed by tumor biology; however, the extent of damage and success of subsequent regeneration can be influenced by the age of the host. Older patients may experience greater net bone loss and reduced regenerative capacity, consistent with age-related changes in bone and immune function. Therapeutically, this necessitates a combination of oncological control and regenerative strategies tailored to the aged bone environment.

## Immunomodulatory strategies for bone regeneration

Given the interplay between the immune system and bone cells, new therapeutic strategies are increasingly focusing on immunomodulation to enhance bone regeneration, especially in compromised scenarios such as aging or chronic inflammation. Conventional bone-regenerative approaches in dentistry have relied on graft materials, barrier membranes for guided tissue regeneration, and growth factors such as bone morphogenetic protein-2 or platelet-derived growth factor. Although these address osteogenic and scaffold aspects, they do not directly address the inflammatory microenvironment, which is often hostile in aged or diseased tissues. Immunomodulatory strategies aim to tilt the immune response from a pro-resorptive chronic inflammatory state toward a pro-regenerative resolved state that supports healing.

### Targeting inflammatory cytokines and pathways

One approach involves the use of agents that neutralize key cytokines that drive bone loss. For instance, blocking IL-1β or TNF-α with specific inhibitors has been explored in rheumatologic conditions and could theoretically benefit periodontal or periapical healing. In animal models of periodontitis, the inhibition of TNF resulted in reduced alveolar bone loss, confirming that these cytokines are valid targets. However, broad immunosuppressive approaches may result in adverse effects. One example is the inhibition of the NLRP3 inflammasome. A small-molecule inhibitor of NLRP3, MCC950, suppresses osteoclast differentiation and significantly reduces alveolar bone loss in a ligature-induced periodontitis model. By preventing inflammasome assembly, MCC950 curbed the downstream cascade of inflammation-driven osteoclastogenesis [47]. Such an approach could be particularly beneficial in aging individuals in whom NLRP3 may be overactive. Another related strategy is targeting downstream effectors such as caspase-1 or IL-1β directly. Indeed, anakinra (an IL-1 receptor antagonist) could hypothetically be delivered locally to periodontal pockets to dampen bone-resorptive signals.

Although cytokine blockade represents a biologically rational strategy, local inhibition of IL-1 or related inflammatory mediators in periodontal or periapical tissues remains experimental. Critical issues regarding feasibility, optimal dosing, delivery systems, infection risk, and potential interference with physiological wound healing have not been resolved. At present, such approaches should be regarded as hypothesis-generating concepts supported mainly by preclinical evidence, and they require rigorous translational and clinical evaluation before consideration for routine clinical application.

### Modulating macrophage polarization

Macrophages play pivotal roles in tissue repair. M1-polarized macrophages secrete inflammatory cytokines and prolong tissue damage, whereas M2-polarized macrophages release anabolic growth factors that aid regeneration. Strategies to encourage macrophages to assume an M2 pro-healing phenotype in bone lesions are currently being explored. One approach involves the release of cytokines, such as IL-4 or IL-10 (which drive M2 polarization). The other is the use of microRNAs or small molecules that modulate macrophage signaling. For example, one study reported using a nanostructured hydrogel carrying microRNA-24 mimics, which, when applied to a periodontal defect, shifted macrophages toward an M2 phenotype and improved bone regeneration outcomes [48].

## MSCs and their secretome

MSC-based therapies are inherently regenerative and immunomodulatory. MSCs can differentiate into osteoblasts; however, they also exert paracrine effects on immune cells, such as suppressing T cell proliferation, altering macrophage polarization, and releasing anti-inflammatory cytokines. A systematic review of animal studies reported that the local delivery of MSCs to periodontal defects significantly enhances bone fill and attachment gain, often accompanied by lower inflammatory cell infiltration [49]. In addition to whole-cell therapy, MSC-derived exosomes represent an exciting acellular approach. Exosomes from young, healthy MSCs can be enriched for regenerative signals. Some MSC exosomes also contain angiogenic factors that improve blood vessel formation in the defect, which is crucial for supporting new bones. One advantage for aging patients is that exosomes can be manufactured from young donor cells, which might overcome the intrinsic cellular deficits of aged cells.

### Anti-senescence (senolytic) approaches

Based on the evidence that senescent cells in the bone impede regeneration by secreting deleterious factors, clearing these cells or suppressing their SASP may enhance healing. Senolytic drugs, such as dasatinib and quercetin, have shown promise in improving bone density in aged mice by removing senescent osteocytes [50]. In periodontal disease, one can envision the local delivery of senolytic agents to periodontal pockets to eliminate senescent fibroblasts or osteoblasts that contribute to chronic inflammation. Although these approaches are still largely experimental in the oral arena, they hold great potential, particularly for older patients with chronic refractory periodontitis or those needing improved healing after bone grafting.

However, evidence supporting senolytic or senomorphic strategies in oral tissues, particularly in alveolar bone and periodontal structures, remains very limited. To date, most studies have been focused on long bones or systemic skeletal aging, primarily using murine models, and direct experimental data evaluating the effects of senescent cell clearance on periodontal or periapical bone regeneration are lacking.

A recent mouse study reported DEL-1 as an endogenous senolytic factor capable of reducing cellular senescence and attenuating age-related bone loss in the appendicular skeleton, providing mechanistic support for targeting senescence in skeletal aging [51]. However, these findings were obtained exclusively in murine models and non-oral skeletal sites, and their relevance to the jaw, alveolar bone, and human oral disease remains to be established.

Therefore, senolytic strategies should currently be regarded as exploratory and hypothesis-generating rather than clinically actionable approaches for oral bone regeneration. Rigorous preclinical studies using aged models of periodontitis, periapical disease, and post-surgical jaw healing are required to define efficacy, safety, and therapeutic windows before any consideration of clinical translation in dentistry.

## Future directions

Future work on alveolar bone regeneration in the aging population should move beyond extrapolating data from young, healthy cohorts and instead explicitly integrate aging biology into the study design. A central priority is better phenotyping of the “aged” oral bone microenvironment. This includes the development of clinically applicable biomarkers of osteoimmunological status (e.g., circulating or salivary indices of inflammaging, senescence markers, and inflammasome activity) that can stratify older patients into distinct risk and response profiles for periodontitis, periapical lesions, and tumor-associated bone loss. Parallel efforts are needed to refine experimental models; most periodontal, endodontic, and tumor–bone interaction studies still use young animals, whereas future studies should routinely employ aged models, including those with common comorbidities (diabetes, estrogen deficiency, prior radiation, and antiresorptive therapy), to better mirror real-world geriatric patients.

On the therapeutic side, promising immunomodulatory strategies require rigorous preclinical optimization and careful translational pathways. Targeting of inflammasome pathways, key cytokines, or macrophage polarization must be balanced against infection control, especially at chronically contaminated oral sites. MSC- and exosome-based approaches, as well as senolytic or senomorphic interventions, should be systematically evaluated in aged models of periodontitis and periapical disease, with a focus on delivery routes, dosing windows, and long-term safety. In tumor-related settings, future work should define how to couple oncologic control (including antiresorptives and radiotherapy) with strategies that preserve or restore the regenerative capacity in the irradiated, aged jaw.

Further clinical research is required to provide mechanistic insights. Prospective trials of regenerative and immunomodulatory therapies should intentionally include older adults with subgroup analyzes based on age, frailty, and systemic inflammatory status. Endpoints should extend beyond short-term radiographic filling and include the durability of regeneration, recurrence of disease, oral function, and oral–systemic impacts. Taken together, these directions point toward a more personalized, age-aware regenerative dentistry that treats the alveolar bone not as a passive scaffold, but as an immune-regulated organ whose behavior in late life can, in principle, be favorably modified.

## Conclusion

Aging reshapes bone metabolism and immunity in ways that render the alveolar process more vulnerable to destruction and less capable of repair. Cellular senescence, stem cell exhaustion, immunosenescence, inflammaging, inflammasome activation, and remodeling of the bone marrow niche collectively shift the balance toward bone resorption and impaired regeneration. In periodontitis, periapical lesions, and tumor-associated bone loss, these age-related mechanisms amplify or distort host responses to microbial and neoplastic insults, contributing to a “high-inflammation, low-repair” phenotype in older patients.

Meanwhile, age is a powerful modifier rather than a deterministic cause: many older individuals maintain stable alveolar bone when inflammatory and oncologic challenges are controlled. In this review, we highlight that effective management of alveolar bone loss in older adults requires more than mechanical debridement, conventional grafts, or oncologic resection alone. Integrating immunomodulatory strategies, senescence-targeted approaches, and advanced biomaterials with age-appropriate diagnostics and models offers a realistic approach for improving outcomes. In this review, we focused on aging-related biological mechanisms and we did not attempt to quantitatively disentangle the individual contributions of comorbidities, lifestyle factors, or pharmacologic exposures, which variably modify alveolar bone outcomes in real-world clinical populations. As the population ages, the ability to regenerate and maintain alveolar bone in late life is central to preserving oral function, nutrition, and quality of life. Therefore, understanding and therapeutically harnessing the intersection of bone biology and immunology in aging is a scientific challenge and a clinical imperative for future regenerative dentistry.
